# Comparison of Suprachoroidal Drug Delivery with Subconjunctival and Intravitreal Routes Using Noninvasive Fluorophotometry

**DOI:** 10.1371/journal.pone.0048188

**Published:** 2012-10-31

**Authors:** Puneet Tyagi, Rajendra S. Kadam, Uday B. Kompella

**Affiliations:** 1 Nanomedicine and Drug Delivery Laboratory, Department of Pharmaceutical Sciences, University of Colorado Anschutz Medical Campus, Aurora, Colorado, United States of America; 2 Department of Ophthalmology, University of Colorado Anschutz Medical Campus, Aurora, Colorado, United States of America; 3 Department of Bioengineering, University of Colorado Anschutz Medical Campus, Aurora, Colorado, United States of America; National Eye Institute, United States of America

## Abstract

**Purpose:**

To determine whether exposure of sodium fluorescein (NaF) to the choroid-retina region in the posterior segment of the eye is greater with suprachoroidal injection when compared to intravitreal and transscleral routes.

**Methods:**

Suprachoroidal injection, a new approach for drug delivery to the posterior segment of the eye was validated using a 34 G needle and Indian ink injections in Sprague Dawley rats, followed by histology. Delivery of NaF was compared in Sprague Dawley rats after suprachoroidal, posterior subconjunctival, or intravitreal injections. NaF levels were monitored noninvasively up to 6 hours using Fluorotron Master™, an ocular fluorophotometer Pharmacokinetic parameters were estimated using WinNonlin.

**Results:**

Histological analysis indicated localization of India ink to the suprachoroidal space below sclera, following injection. NaF delivery to choroid-retina was in the order: suprachoroidal > intravitreal >posterior subconjunctival injection. Peak NaF concentration (C_max_) in choroid-retina was 36-fold (p = 0.001) and 25-fold (p = 0.001) higher after suprachoroidal (2744±1111 ng/ml) injection when compared to posterior subconjunctival (76±6 ng/ml) and intravitreal (108±39 ng/ml) injections, respectively. NaF exposure (AUC_0–360min_) to choroid-retina after suprachoroidal injection was 6-fold (p = 0.001) and 2-fold (p = 0.03) higher than posterior subconjunctival and intravitreal injections, respectively. Choroid-retina T_max_ was observed immediately after dosing with suprachoroidal injections and at 10 and 27.5 minutes, respectively, with subconjunctival and intravitreal injections.

**Conclusions:**

Suprachoroidal injections are feasible in a rat model. Suprachoroidal injections resulted in the highest bioavailability, that is, the extent and rate of delivery of NaF to choroid-retina, when compared to intravitreal and posterior subconjunctival injections. Ocular fluorophotometry is useful for noninvasive monitoring of NaF in rats following administration by various routes including suprachoroidal route.

## Introduction

Diseases of the posterior segment of the eye are responsible for severe vision loss and blindness in the developed countries. The most prevalent posterior segment diseases include age related macular degeneration (AMD), diabetic retinopathy, and retinal degenerative diseases. As of 2008, AMD is prevalent in 8 million in the USA and is expected to increase to 12 million by 2020 [1]. Nearly 10% of the subjects suffering from AMD are diagnosed with the growth of abnormal or leaky blood vessels in the choroid below the retina, a condition known as wet AMD or choroidal neovascularization (CNV). CNV is primarily responsible for significant loss of vision and blindness in AMD patients. Diabetic retinopathy is prevalent in 4.1 million people in the United States, with nearly 22% (0.9 million) of diabetic patients having vision threatening diabetic retinopathy [2]. Further, the number of diabetic patients in the USA is expected to rise to 16 million by 2050 [2]. Increase in prevalence of these vision threatening disorders is also resulting in a rise in the cost of treatment [3]. Despite the severity and increasing prevalence of back of the eye diseases, conventional drug delivery methods are either inefficient in delivering required amount of drug to the site of action or highly invasive to the vitreous humor, with significant side effects.

The most common drug delivery method for treating ocular disorders is topical administration, primarily due to its convenience. Unfortunately, topically administered treatments are rapidly drained from the ocular surface, resulting in less than 5% bioavailability, that too mainly to the tissues in the anterior segment of the eye [4]. Due to the barriers present, currently there is no eye drop formulation approved for treating back of the eye diseases. To bypass the barriers associated with topical delivery for back of the eye diseases, intravitreal injections are becoming popular [5], [6]. However, intravitreal injections are highly invasive and associated with complications such as cataract, retinal detachment, vitreous hemorrhage, and endophthalmitis [7]. Other than topical and intravitreal routes of delivery, periocular routes such as sub-Tenon and subconjunctival routes can also be used to deliver drugs to the posterior segment of the eye [8], [9]. The periocular routes place the therapeutic agent adjacent to the sclera for transscleral delivery, thereby reducing the risks associated with the intravitreal route of administration [10]. Nevertheless, periocular routes have disadvantages such as hemorrhage at the site of injection [11], [12]. Thus, development of a safe and efficacious route of delivery for the treatment of posterior segment disorders remains the foremost challenge in ocular drug delivery research.

Suprachoroidal space (SCS) [13] is a unique, anatomically advantageous space that localizes therapeutic agents adjacent to the choroid-retina region, the target tissue affected in the neovacular form of age related macular degeneration and diabetic retinopathy. Safety of injections into the SCS was shown by Einmahl et al. [14], wherein a novel poly (ortho ester) biomaterial was evaluated, and by Poole et al., [15] wherein sodium hyaluronate was injected to treat retinal detachments. Einmahl et al., [14] observed that poly (ortho ester) injection in the SCS caused no clinical complications except some slight choroidal pigmentation and presence of vacuoles in the SCS. Poole et al., [15] observed slight bleeding and inflammation at the site of injection, which disappeared within 3 weeks. Olsen et al. [16] evaluated the safety of a novel cannula system for delivery in the SCS by monitoring histopathology and retinal and choroidal blood flow in monkeys and pigs and observed minor tissue injury at the site of injection. More recently, Patel et al. [17] developed and evaluated a minimally invasive strategy using a novel hollow microneedle system to study the ex vivo suprachoroidal distribution of sulforhodamine B dye and particles ranging in size from 20 to 1000 nm. Suprachoroidal delivery is minimally invasive and might be safer because it does not require entry into the vitreous, thereby potentially protecting retina from any injection related damage.

Even though suprachoroidal delivery is being evaluated for effective treatment of posterior segment disorders, there are no reports comparing it to periocular injections. Further, there are limited investigations comparing suprachoroidal and intravitreal routes of delivery, that too for a protein drug but not small molecules [18]. Since choroid vessels have high blood flow, it is generally perceived that drug molecules can clear very rapidly. Therefore, a direct comparison of different routes of drug administration will help establish the relative advantage of suprachoroidal delivery.

We used a non-invasive ocular fluorophotometry technique to study the distribution of NaF following different routes of injection. Following periocular injections, a few pharmacokinetics studies have been conducted using ocular fluorophotometry for small molecules such as NaF [19] and oregon green–labeled triamcinolone acetonide [20] and macromolecules such as high molecular weight FITC-dextran (40 kDa and 70 kDa) [21]. Traditional methods of evaluating ocular pharmacokinetics are invasive and costly. Sacrificing animals at multiple time points followed by eye enucleation and isolation of different ocular tissues makes the process tedious and time consuming. Further, changes in drug location and concentration can occur during tissue extraction. In comparison, ocular fluorophotometry is a non-invasive technique, which does not affect ocular tissues and allows time course evaluations in the same animal in different ocular tissues using a single scan. In this study, we determined the delivery and pharmacokinetics of NaF injected in suprachorodial space of rats and compared it with intravitreal and posterior subconjunctival injections using ocular fluorophotometry. NaF is a rational choice for in vivo fluorophotometry because of its safety [22], high absorptivity, and fluorescence yield [23]. Further, the molecular weight of NaF (376 Da) is similar to many antimicrobial agents and steroids administered to the eye for the treatment of ocular disorders.

## Materials and Methods

### Materials

Sodium fluorescein (NaF) used in this study was purchased from Sigma-Aldrich (St. Louis, MO).

### Ethics Statement

All animals were treated according to the Association for Research in Vision and Ophthalmology (ARVO) statement for the Use of Animals in Ophthalmic and Vision Research. Animal protocols followed during this study were approved by the Institutional Animal Care and Use Committee of the University of Colorado Anschutz Medical Campus, Aurora, CO.

### Administration of NaF by Different Routes

Adult male Sprague Dawley (SD) rats (150–180 g) were purchased from Harlan Sprague Dawley Inc. (Indianapolis, IN, USA). Rats were anesthetized using an intraperitoneal injection of a mixture of 80 mg/kg ketamine and 10 mg/kg xylazine. Using a 10 µl Hamilton glass syringe (Hamilton company, NV) fitted with a 34 gauge needle (1/2 inch long; with a 45^o^ taper), 5 µl phosphate buffer saline (PBS; pH 7.4) containing 100 µg/ml NaF was injected into the suprachoroidal space, posterior subconjunctival region, or vitreous humor of the right eye of the rat.

### Histology of Rat Eye after Suprachoroidal Injection

To confirm the accuracy of suprachoroidal injection in vivo in rat eyes, histological examination of rat eye after suprachoroidal injection of India ink dispersion was performed. Rats were anaesthetized and 5 µl of 5% India ink dispersion was injected in the suprachoroidal space using a 10 µl Hamilton glass syringe fitted with a 34 gauge needle (1/2 inch long; with a 45^o^ taper). Rats were immediately euthanized and eyes enucleated. Eyes were further fixed in 4% formalin solution for 2 days. Paraffin sections (5 um thick) were obtained and stained with haematoxylin and eosin. Sections were observed under a light microscope (Olympus BX41 laboratory microscope) fitted with a camera (Diagnostics instruments Inc.).

### Ocular Fluorophotometry

The disposition of NaF was studied using Fluorotron Master™, an ocular fluorophotometer (OcuMetrics Inc., Mountain View, CA) fitted with a small animal adapter. The Fluorotron scans report NaF concentrations in ocular tissues at 0.25 mm intervals along an optical axis used by the instrument. Prior to acquiring Fluorotron scans, a single drop of 1% tropicamide (Mydriacyl, Alcon laboratories, Inc., TX) solution was instilled in the eyes. Baseline fluorescence was measured prior to NaF injections. After NaF injections, Fluorotron scans were acquired up to six hours, with repeated application of tropicamide drops every 2 hours. The maximum pupil diameter size (pupillary dilation) with 1% tropicamide solution eye drops is attained within ∼ 40 minutes [24] and persists up to ∼ 70 minutes, requiring repeated instillation. Saline solution eye drops were applied to the eyes periodically, to prevent dehydration of the corneas. The raw data from the fluorophotometer was transferred to a spreadsheet (Excel; Microsoft Corporation, WA) and plotted.

### Pharmacokinetic and Statistical Analysis

Non-compartmental pharmacokinetic analysis for the three routes of injection was performed using WinNonlin software (Version 1.5, Scientific Consulting, Inc.). AUC_0–360 min_ is the area under the curve obtained by plotting the concentration-time data, where‘t’ is the last time point at which NaF levels were measured. The “t” value was 360 minutes for the three routes of administration. The 0-time point concentration was considered as zero when the drug was measured away from the site of dosing (extravascular dose mode in WinNonlin). When the drug was measured at the site of administration (e.g., estimation of choroid levels after suprachoroidal injection or vitreal levels after intravitreal injection), WinNonlin estimated the 0-time concentration by extrapolating the data to y-axis. A statistical comparison of the pharmacokinetic parameters was performed using one-way ANOVA followed by Tukey's post hoc analysis (SPSS, ver.11.5; SPSS, Chicago, IL). The results were considered statistically significant at p<0.05.

## Results

### Histology of Rat Eye after Suprachoroidal Injection

**Figure 1:**
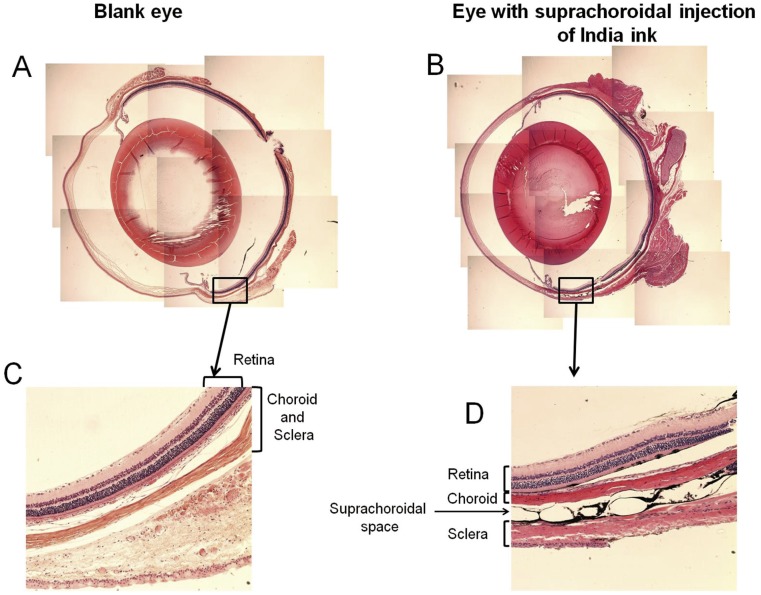
Suprachoroidal injection of India ink between sclera and choroid-retina in SD rats. Eyes were injected with 5 µl of 5% India ink dispersion in suprachoroidal space. The eyes were fixed in 4% formalin and embedded in paraffin blocks. H & E stained sections were examined. (A) A 4× magnification image showing a cross section of a blank eye; (B) Eye administered with suprachoroidal injection; (C) 10× magnification of a blank eye; and (D) A 10× magnification of the site of suprachoroidal injection showing the presence of India ink in the suprachoroidal space.

Since this was the first study to evaluate the pharmacokinetics of NaF after suprachoroidal injection in rats, the accuracy of the suprachoroidal injection was confirmed by histological sectioning of India ink injected SD rat eyes (Figure 1). The histological cross section of India ink injected SD rat eyes showed a spread of India ink between the sclera and choroid. Suprachoroidal injection resulted in widening of suprachoroidal space as compared to control eyes (Figure 1D), which might be due to the pressure created by the India ink injection. Similar widening of suprachoroidal space was also observed by Patel et al. [17]. SD rat eyes without any injection of India ink were used as the negative control, which showed no black color in any part of the eye (Figures 1A and 1C).

### Fluorophotometric Measurement

The Fluorotron Master is calibrated to provide readouts of fluorescence in NaF concentrations. Thus, readings from the scans were directly used as NaF concentrations in a given region of the eye. In the Fluorotron Master a blue excitation light is delivered through the optics of the system to the eye and the resulting emitted fluorescent light is collected via the same optical system. A measurement area is created at the point where the excitation and emission lights intersect and is known as the focal diamond [25]. The focal diamond, a measure of resolution inside the rat eye, is 400 µm. Levels of fluorescence are measured within this focal diamond, and the focal diamond is automatically moved along the axis of the eye in the posterior to anterior direction.

**Figure 2:**
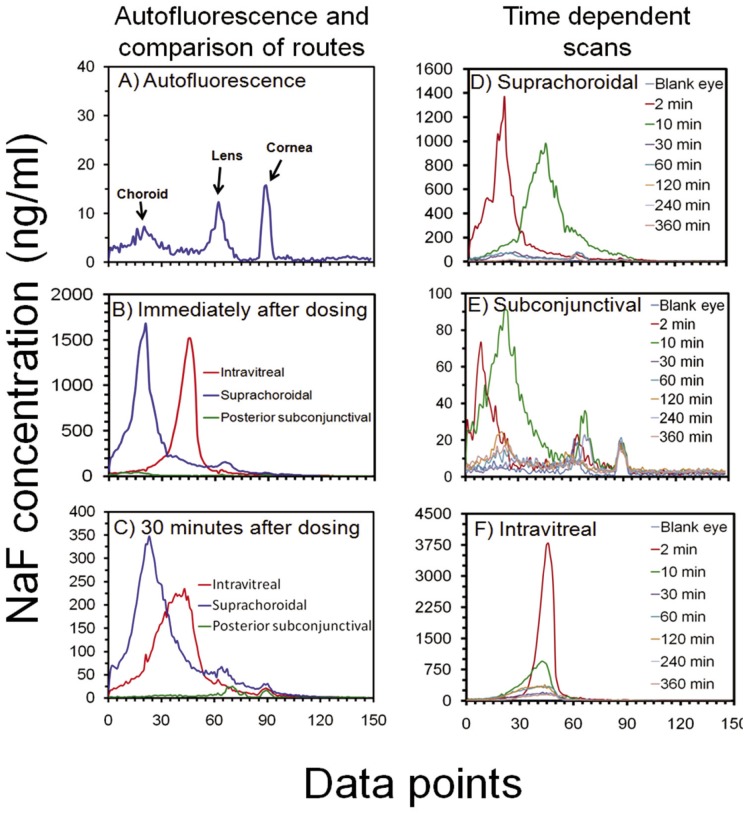
Representative fluorophotometry scans attained using Fluorotron Master™ in Sprague Dawley rat eye. Scans are for (A) blank eye showing autofluorescence, (B) eyes immediately after injection of NaF in suprachoroidal, posterior subconjunctival, or vitreous region, (C) eyes 30 minutes after injection of NaF in suprachoroidal, posterior subconjunctival, or vitreous region. Data in panel A is an average for n = 6, and in B and C it is an average for n = 4. Representative time dependent scans after injection of NaF in (D) suprachoroidal, (E) posterior subconjunctival, and (F) vitreous regions are also shown. Blank eye scan shows the autofluorescence of choroid-retina, lens, and cornea regions.

Following the above protocol, we obtained scans for blank eyes and eyes injected with NaF by different routes. NaF concentrations in the eye were plotted against distance data points separated by 0.25 mm on an optical axis. This distance in millimeters on the plot cannot be related to the actual dimensions of rat eye tissues. However, potential tissue assignments to data points can be made based on tissue autofluorescence peaks and by monitoring fluorescence signal following sodium fluorescein injections in various compartments of the eye. Figure 2 shows representative Fluorotron scans for blank eyes (Figure 2A) and eyes injected with NaF by suprachoroidal, posterior subconjunctival, and intravitreal routes immediately (Figures 2B) and 30 minutes after injection (Figure 2C).

Based on the known ability of choroid, lens, and cornea to autofluoresce, data points at approximately 25, 61, and 88 in baseline Fluorotron scans were considered as autofluorescence peaks corresponding to choroid, lens, and cornea, respectively. Following NaF injections, peak signals for posterior subconjunctival, suprachoroidal, and intravitreal injections were evident at data points 10, 21, and 45, respectively, in the Fluorotron scans (Figure 2D–E). The vitreal peak at 45^th^ data point (Figure 2F) was also observed by Ishiko et al. during a fluorescein distribution study in a tree shrew [26]. The blank eye scans (Figure 2A) (n = 6) showed 6.5 (±5.53), 12.3 (±7.5), and 15.7 (±3.2) ng/ml autofluorescence measured as sodium fluorescein equivalents in choroid, lens, and cornea regions, respectively. The autofluorescence for the anterior chamber in the valley between lens and cornea peaks at data point 77 was 0.5±0.31 ng/ml (n = 6). Therefore, NaF concentrations in dosed animals at data points 21, 45, and 77 were assigned as concentrations in choroid-retina, vitreous, and, anterior chamber respectively, for pharmacokinetic analysis.

### NaF Pharmacokinetics after Suprachoroidal Injection

**Figure 3:**
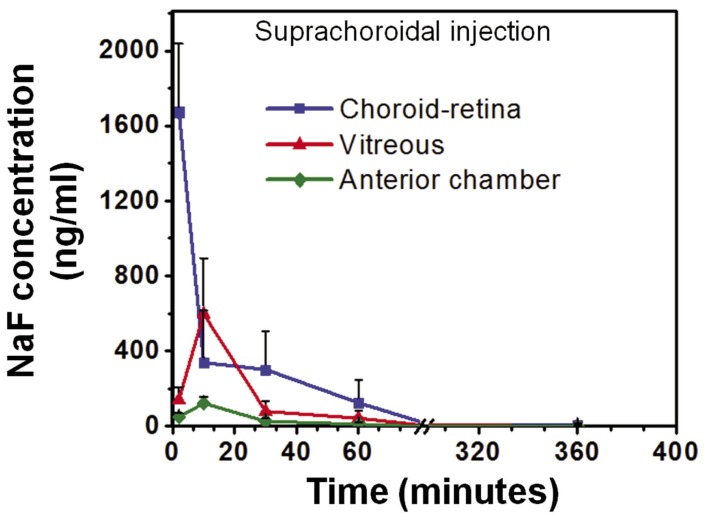
Sodium fluorescein concentrations in choroid-retina, vitreous, and anterior chamber regions after injection in the suprachoroidal space. At 2 minutes after the injection, the choroid-retina had the highest concentration (1673±363 ng/ml). Vitreous and anterior chamber regions had peak concentrations of 597±297 ng/ml and 122±34 ng/ml, respectively, at 10 minutes. The baseline values for choroid, vitreous, and anterior chamber were 6.5 (±5.53), 1.65 (±0.78), 0.5 (±0.31) ng/ml, respectively. Data is presented as mean ± S.D. for n = 4.

Figure 3 shows the mean NaF concentration in different regions of the eye after suprachoroidal injection of NaF. The amount of fluorescein in the choroid-retina region observed immediately after the injection was 1673±363 ng/ml (at 2 minutes). The concentrations extrapolated to time zero by WinNonlin were 2744±1111 ng/ml. The peak fluorescein concentrations in the vitreous and the anterior chamber regions were 597±297 and 122±34 and ng/ml, and 3- and13- fold lower, respectively, than the choroid-retina region. The concentration of fluorescein peaked at 10 minutes in all animals in the vitreous as well as the anterior segment regions.

### NaF Pharmacokinetics after Subconjunctival Injection

**Figure 4:**
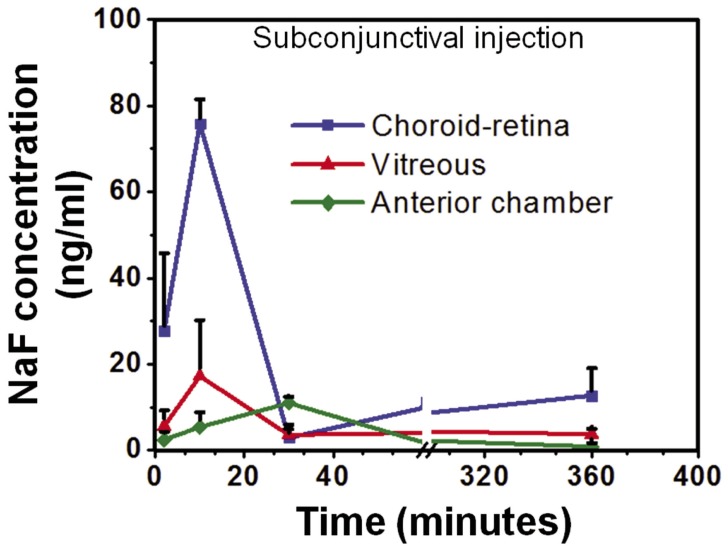
Sodium fluorescein concentration in choroid-retina, vitreous, and anterior chamber regions after posterior subconjunctival injection. At 10 and 67.5 minutes after injection, the choroid-retina and vitreous regions had the highest concentrations of 76±6 and 17±13 ng/ml, respectively. Anterior chamber region had a peak concentration of 11±1 ng/ml at 30 minutes. The baseline values for choroid, vitreous, and anterior chamber were 6.5 (±5.53), 1.65 (±0.78), 0.5 (±0.31) ng/ml, respectively. Data is presented as mean± S.D. for n = 4.

Mean NaF concentrations in choroid-retina, vitreous, and anterior segment regions after posterior subconjunctival injection are shown in Figure 4. The peak concentrations in the choroid-retina and vitreous regions observed at 10±0 and 67.5±115 minutes (3 animals had peak at 10 minutes and one exhibited a peak at 240 minutes) were 76±6 and 17±13 ng/ml, respectively. Anterior chamber region showed peak fluorescein value of 11±1 ng/ml at 30±0 minutes.

### NaF Pharmacokinetics after Intravitreal Injection

**Figure 5:**
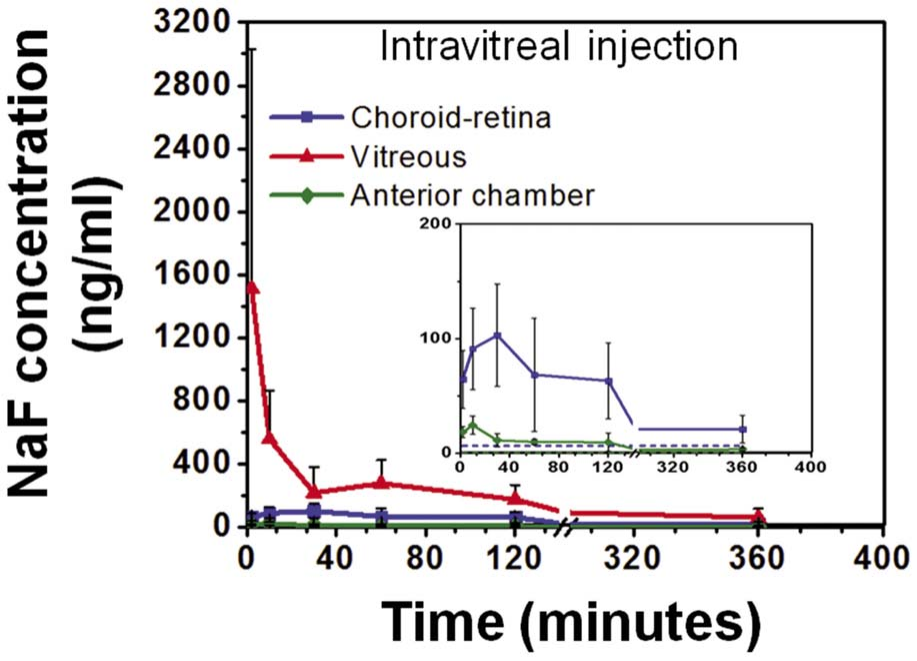
Sodium fluorescein concentrations in choroid-retina, vitreous, and anterior chamber regions after intravitreal injection. At 2 minutes of injection, the vitreous region had the highest concentration (1512±1517 ng/ml). Choroid-retina and anterior chamber regions had peak concentrations of 103±45 ng/ml and 24±8 ng/ml, respectively, at 27.5 minutes and 10 minutes. The baseline values for choroid, vitreous, and anterior chamber were 6.5 (±5.53), 1.65 (±0.78), 0.5 (±0.31) ng/ml, respectively. Data is represented as mean ± S.D. for n = 4. Inset shows NaF levels in choroid-retina and anterior chamber.

Figure 5 shows the mean values for NaF concentration in choroid-retina, vitreous, and anterior chamber regions after intravitreal injection of NaF, with the peak vitreous concentrations measured at 2 minutes being 1512±1517 ng/ml. The concentrations extrapolated to time zero were 2004±2268 ng/ml. The highest NaF concentration (103±44 ng/ml) in the choroid-retina region was observed at 27.5±23.6 minutes and it was 15- fold lower compared to the peak values observed in the vitreous. The anterior chamber region had a peak concentration of 24±8 ng/ml at 10±0 minutes.

### Comparison of Pharmacokinetic Parameters and Drug Concentrations between Different Routes for Choroid-retina Delivery

**Figure 6:**
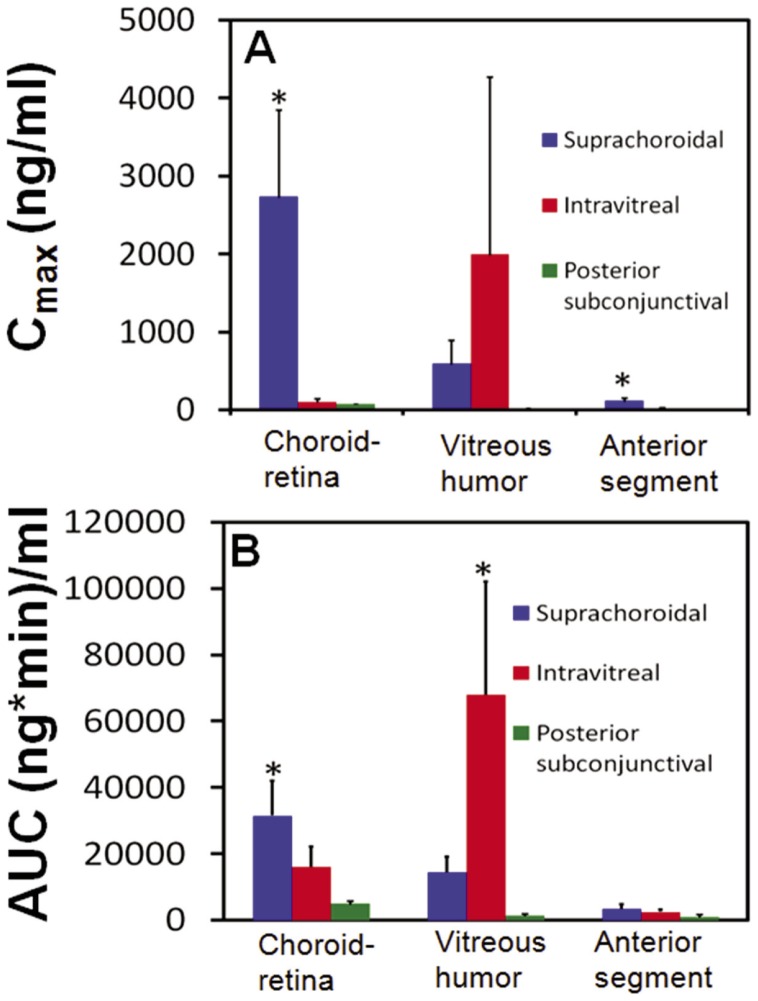
Pharmacokinetic parameters (C_max_ and AUC _0–360 min_) estimated for sodium fluorescein after injection by suprachoroidal, intravitreal, and posterior subconjunctival routes in Sprague Dawley rats. Parameters for the three routes of administration were estimated using non-compartmental analysis using WinNonlin (version 1.5, Pharsight Inc.,CA). C_max_ is the maximum observed drug concentration and AUC_ 0–360 min_ is the area under the curve in a given tissue. Data are expressed as mean ± SD for n  = 4. * indicates p<0.05 compared to other two groups.

C_max_ and AUC_0–360 min_ data is shown in Figure 6. The area under curve (AUC_0–360 min_) for NaF concentration in the choroid-retina region was 6-fold (p = 0.001) and 2-fold (p = 0.03) higher than posterior subconjunctival and intravitreal injections, respectively. The C_max_ of choroid-retina region was 36-fold (p = 0.001) and 25-fold (p = 0.001) higher after suprachoroidal injection compared to subconjunctival and intravitreal injections, respectively. The time to attain maximum concentration (T_max_) in choroid-retina region was in the order: suprachoroidal injection < subconjunctival injection < intravitreal injection. T_max_ for vitreous region was in the order: intravitreal injection < suprachoroidal injection < subconjunctival injection. T_max_ for anterior chamber was 10 minutes for suprachoroidal and intravitreal routes and 30 minutes for subconjunctival route.

At 2 minutes, choroid-retina levels were significantly higher (p<0.05) after suprachoroidal injection when compared to intravitreal and subconjunctival injections. At 30 minutes, choroid-retina levels were significantly higher (p<0.05) after suprachoroidal injection when compared to subconjunctival injection. At 60 minutes, choroid-retina levels were significantly higher (p<0.05) after intravitreal injection when compared to subconjunctival injection. At 120 minutes, choroid-retina levels were significantly higher (p<0.05) after intravitreal injection when compared to suprachoroidal and subconjunctival injections.

At 10, 60, 120, and 240 minutes, vitreous levels were significantly higher (p<0.05) after intravitreal injection when compared to subconjunctival and suprachoroidal injections. At 10 and 30 minutes, vitreous levels were significantly higher (p<0.05) after suprachoroidal injection when compared to subconjunctival injection.

At 2, 30, and 60 minutes, anterior chamber levels were significantly higher (p<0.05) after suprachoroidal injection when compared to subconjunctival injection. Anterior chamber concentrations were significantly higher (p<0.05) after intravitreal injection when compared to subconjunctival injection at 2, 10, 30, and, 60 minutes.

## Discussion

This is the first study to demonstrate suprachoroidal injection in a rat model and compare the pharmacokinetics of suprachoroidal injection with intravitreal and posterior subconjunctival injections using noninvasive ocular fluorophotometry. We demonstrated that 1) sodium fluorescein levels can be monitored noninvasively in different ocular tissues after suprachoroidal, posterior subconjunctival, and intravitreal injections in rats using ocular fluorophotometry; 2) the suprachoroidal route is the most effective method for attaining high concentrations of sodium fluorescein in the choroid-retina region; and 3) the rate and extent of delivery to the choroid-retina is highest with suprachoroidal injection.

### Possible Reasons for Autofluorescence and Broad vs. Sharp NaF Peaks in Different Regions

Baseline Fluorotron scans showed very minimal autofluorescence peaks in the choroid-retina, lens, and cornea regions (Figure 2A). A very low autofluorescence was also observed in the anterior chamber. Possible reasons for autofluorescence from these tissues are the presence of fluorescent nucleotides and lipid metabolites [27]–[29]. Autofluoresence in the choroid-retina region of rats is attributed to the presence of lipofuscin granules [27], [30] in the retinal pigment epithelial cells and elastin layer in the bruch’s membrane [28]. Autofluoresence in the lens can be due to the presence of flavoproteins such as FMN in the lens epithelium [31]. Rat corneal autofluorescence is caused by pyridine nucleotides such as nicotinamide adenine dinucleotide phosphate (NADPH) [32] and flavin nucleotides such as flavin mononucleotide (FMN) [33] in metabolically active cells such as the corneal epithelium and endothelium [29]. Baseline autofluorescence and peak assignments are shown in Figure 2A.

Using fluorophotometry, we compared NaF levels in the eye after suprachoroidal, subconjunctival, and intravitreal injections. The signals observed were much higher than the background fluorescence and each route resulted in peak signals at a distinct location, corresponding to the site of injection. Suprachoroidal injection of NaF in the rat eye showed a broad peak (Figure 2B) possibly due to the ‘halation’ of the choroid-retina response [34]. Halation or secondary fluorescence occurs due to the presence of a highly autofluorescent tissue such as choroid near the point of quantification. Light passing straight through the choroid- retina is reflected back by the choroid base and scattered around. This causes the fluorescence to bleed through and results in tailing of the choroid-retina response. Similar to suprachoroidal injection, the peak attained after subconjunctival injections was also broad (Figure 2C). In the case of intravitreal injections, a comparatively sharper peak was attained (Figure 2D), primarily due to the lack of any secondary fluorescence.

### Higher Rate and Extent of Delivery to Choroid-retina after Suprachoroidal Injection

#### NaF levels in the anterior chamber

We compared the pharmacokinetics of NaF after suprachoroidal, subconjunctival, and intravitreal injections (Figure 3,4,5,6). In pharmacokinetic analysis, the rate and extent of delivery is related to C_max_ and AUC. Further, T_max_ is related to the rate of delivery, if the elimination remains the same in a given tissue, irrespective of the route of drug entry and concentration. Rapid absorption into the choroid-retina region, as indicated by the T_max_ value, was observed immediately upon suprachoroidal injection. In comparison, intravitreal and subconjunctival injections had C_max_ at 27.5 and 10 minutes, respectively. After suprachoroidal injection, the C_max_ of NaF in choroid-retina was 36- and 25- fold higher than subconjunctival and intravitreal injection, respectively. Further, the extent of delivery was also 6- and 2- fold higher than subconjunctival and intravitreal injections, respectively. The higher rate and extent of delivery to choroid-retina after suprachoroidal injection is due to targeted deposition of the dose in the choroid. In comparison to suprachoroidal injection, drug molecules administered by intravitreal and subconjunctival routes need to cross various barriers to reach the target tissue. Olsen et al. [18] have also shown localized delivery to choroid-retina following suprachoroidal injection of bevacizumab.

Araie and Maurice [35] froze and sectioned eyes to expose the vitreous and fixed them to a cryotome stage. The entire vitreous was scanned using a fluorometer. Readings obtained by the fluorometer were normalized by assigning a maximum value behind the lens at 100. Lines were drawn for the values 90, 80, and so on, obtained by interpolation between the measured values. A concentration contour map was created based on the measured values. Araie and Maurice observed that NaF concentration was the highest in the vitreous immediately adjacent to the lens and dropped to a low value of 30 over the entire surface of the retina and iris-ciliary body indicating that the majority of NaF was cleared from the anterior segment [35]. Thus, following intravitreal injections, NaF is expected to predominantly clear through the anterior segment [36]. Therefore, NaF detected in the choroid-retina region after intravitreal injection in our study can be due to accumulation of NaF at the retinal surface [35] and may not truly depict delivery to the choroid-retina region.

Posterior subconjunctival injection in rats, analogous to sub-Tenon injection in humans, is expected to deliver drugs to the back of the eye tissues [10]. The extent of delivery to choroid-retina after subconjunctival injection is lower compared to suprachoroidal route in our study, possibly due to multiple clearance pathways. In comparison to subconjunctival injection, suprachoroidal injection places the entire dose in close proximity to the choroid, thereby resulting in higher drug levels and exposure to the choroid-retina region. Following subconjunctival injection, the drug may encounter several elimination pathways including episcleral and conjunctival vasculature prior to entering the choroid [37].

#### NaF exposure to the vitreous

Both C_max_ and AUC for vitreous humor delivery of NaF were in the order: intravitreal injection > suprachoroidal > subconjunctival. This rank order is consistent with the proximity of vitreous to the site of administration. The further removed the dose was from the vitreous, the lower the drug delivery.

#### NaF exposure to anterior chamber

In our study, we detected very low levels of NaF in the anterior chamber region after suprachoroidal, subconjunctival, or intravitreal injection when compared to NaF levels in other tissues. Following suprachoroidal injection, anterior chamber C_max_ was significantly higher than intravitreal injection and subconjunctival injection, with the rank order being: suprachoroidal > intravitreal > subconjunctival. A similar rank order was observed for NaF exposure in the anterior chamber. Contrary to our observations, following suprachoroidal injections in ex vivo porcine eyes, Seiler et al. [38] could not detect any signal for contrast agent in the anterior segment region. This may be because following suprachoroidal and subconjunctival injections [37], clearance occurs immediately due to the proximity of blood vessels when compared to intravitreal injections. Therefore, very low quantities of NaF reach the anterior chamber after suprachoroidal and subconjunctival injections. The suprachoroidal injections in our study may be more anterior compared to earlier studies, resulting in significant NaF exposure to the anterior segment. Additionally, the sensitivity of detection of contrast agents may not have been sufficient in the earlier study [38] to pick up the signal from the anterior segment following suprachoroidal injection. Future studies need to assess the influence of site of suprachoroidal injection on drug distribution.

#### NaF clearance by various routes

Although the half-lives for the terminal declining phase of concentration-time profiles could not be estimated for various tissues due to fluctuations in the signal in the terminal regions, the time for NaF levels to approach baseline values in choroid-retina was in the following rank order: intravitreal > suprachoroidal > subconjunctival. While the rapid approach to baseline with subconjunctival route can be attributed to lowest drug delivery by this route, slow approach to baseline with intravitreal route is most likely due to slow absorption of the drug to the choroid from the vitreous humor. NaF in choroid is expected to be eliminated by the same pathways irrespective of the mechanism of drug entry/administration. Also, the elimination kinetics are expected to be the same irrespective of the route of administration, unless the elimination pathways are affected by drug concentrations. Once in the choroid, NaF be removed rapidly due to choroidal blood flow. Rapid drug clearance from choroid is empirically attributed to high blood flow. The blood flow velocity in the human choroid (1–1.2 ml/min; 0.052–0.198 m/s [39]) is several fold lower than the blood flow (1175–2110 ml/min [40]; 0.585–0.766 m/s [41]) in the liver, a primary organ for drug clearance. However, after tissue weight normalization the choroidal blood flow is significantly higher than the hepatic blood flow. Previous studies indicated that tissue weight normalized blood flow to the human choroid and liver were 1200 ml/100 gm tissue/min [42] and 1.7 ml/100 gm/min [43], respectively. Thus, although the total blood flow per unit time and the velocity of the blood in choroid are much lower compared to the liver, the blood supply per unit tissue weight is much higher in the choroid than the liver. However, it is unclear how these differences in blood flow play a role in choroid clearance of solutes. For liver clearance of drugs, total blood flow is taken into consideration [44]. Given the much lower total blood flow in the choroid, it is anticipated that the clearance in choroid would be much less compared to the liver, especially for drugs with high extraction ratio.

In summary, this study shows that the suprachoroidal injection is the most effective route for localized delivery of therapeutics to the choroid-retina region. Further, in this study we have also demonstrated the applicability of ocular fluorophotometry for non-invasive monitoring of drug levels following administration by various routes. However, one of the limitations of ocular fluorophotometry is that this technique cannot be used for drug molecules that are not fluorescent similar to fluorescein. Therefore, most drug molecules require a fluorescein-like tag to be monitored by fluorophotometry. However, such tags may alter physicochemical properties of small solutes and drugs, thereby potentially altering their rate and/or extent of delivery to the eye tissues.
